# Yoga at Every Size: A Preliminary Evaluation of a Brief Online Size-Inclusive Yoga and Body Gratitude Journaling Intervention to Enhance Positive Embodiment in Higher Weight College Women

**DOI:** 10.3389/fgwh.2022.852854

**Published:** 2022-05-26

**Authors:** Jennifer B. Webb, Meagan P. Padro, Erin Vinoski Thomas, Alexandria E. Davies, Lena Etzel, Courtney B. Rogers, Natalia I. Heredia

**Affiliations:** ^1^Department of Psychology, University of North Carolina at Charlotte, Charlotte, NC, United States; ^2^Department of Education, UNC Chapel Hill, Chapel Hill, NC, United States; ^3^Department of Health Policy and Behavioral Sciences, School of Public Health, Georgia State University, Atlanta, GA, United States; ^4^Department of Psychology, Virginia Commonwealth University, Richmond, VA, United States; ^5^Cherokee Health Systems, Knoxville, TN, United States; ^6^Department of Health Promotion and Behavioral Sciences, School of Public Health, University of Texas Health Science Center at Houston, Houston, TX, United States

**Keywords:** yoga, positive body image, embodiment, higher weight, college women, body gratitude writing

## Abstract

The present pilot randomized controlled trial (RCT) evaluated the feasibility, acceptability, and preliminary efficacy of a 4-week online yoga and body gratitude journaling intervention for strengthening positive embodiment among racially-diverse higher weight college women. Seventy-five participants were initially randomized to either the yoga condition (*n* = 36) or to a wait-list control (*n* = 39). Participants completed measures of positive and negative body image, weight bias internalization, self-compassion, drive for leanness, and physical activity acceptance at both baseline and post. Preliminary results among the 42 analyzed completers (mean age = 20.9, *SD* = 2.4; 30% Black or African American) revealed acceptable feasibility given the low-intensity nature of the intervention reflected in a 36% attrition rate. Self-reported adherence was strong for the yoga component with 81% of participants indicating that they practiced with the videos ≥3–4 times per week as suggested. Although 71% reported completing the body gratitude journal ≥1–2 times per week, daily adherence was minimal. Acceptability was also high among participants randomized to the yoga condition as indicated by 86% expressing at least moderate levels of satisfaction with the overall program. Qualitative feedback from participants further supported the acceptability of the program and pointed to important areas in further refining the protocol in the future. Preliminary efficacy was supported by significant reductions in internal body shame and gains in body appreciation, functional body appreciation, functional body satisfaction, functional body awareness, and behavioral commitment to physical activity engagement among the yoga vs. wait-list control participants. These promising findings once replicated in larger, higher-powered trials may have important implications for extending the reach and accessibility of mind-body wellness practices like yoga to benefit racially-/ethnically-diverse college women of higher weight. This research is further responsive to the growing need for efficacious remotely-delivered, and scalable behavioral health interventions in the ongoing era of the COVID-19 pandemic. However, additional research is warranted to explore ways of enhancing engagement of participants with lower levels of positive embodiment and to further incentivize the journaling component of the intervention.

## Introduction

Researchers have recently shifted toward exploring *positive* body image in addition to negative body image, particularly within the contexts of prevention and treatment (1, 2). Effective treatments have the potential to prevent the psychological distress associated with negative body image; however, research supporting the efficacy of various treatments in college-aged women is limited (3).

Yoga has been identified as one intervention that has the potential to reduce negative body image *and* promote aspects of positive body image (3, 4). Cross-sectional research has found that women who practice yoga or self-identify as yoga practitioners are less likely to report body image dissatisfaction, self-objectification, and disordered eating attitudes, and have higher awareness of and responsiveness to bodily sensations and greater body satisfaction than those who do not practice yoga (5–7). Recent experimental research found that college-aged women who participated in a yoga intervention had more positive appearance evaluation, increased body areas satisfaction, and decreased appearance investment compared with wait-list controls (3). Research conducted in predominantly female samples further suggests that yoga interventions that specifically target incorporating an emphasis on body acceptance and gratitude may stimulate more robust gains in positive body image and embodiment [e.g., (8–10)]. Meanwhile, another study found that a 12-week mindful yoga intervention reduced internalized weight stigma in adults (11). Whereas, a systematic review found that yoga can alleviate symptoms in those with disordered eating (12).

Yoga may be a promising intervention for improving body image, but research remains limited in exploring its potential acceptability among more size-diverse groups and in determining the efficacy of various intervention delivery methods within such populations. Indeed, amidst the ongoing era of COVID-19, it has become increasingly critical to develop and evaluate scalable behavioral health interventions that are remotely accessible. Further, research has yet to combine the potential synergistic benefits of yoga practice with body gratitude/functionality appreciation writing. The latter of which has shown significant benefits for improving body image [see (13) for a comprehensive review]. Therefore, to innovatively address these gaps, the present study sought to evaluate the feasibility and acceptability of a 4-week, minimally-guided, online size-affirming yoga and body gratitude writing-based intervention for targeting a range of embodiment-related self-regulatory variables in an ethnically-diverse sample of higher-weight college women. A more exploratory secondary aim was to assess the preliminary efficacy of this brief integrative mind-body approach relative to a wait-list control on the measures of interest (i.e., positive and negative body image, weight bias internalization, acceptance of physical activity-related discomfort, drive for leanness, and, self-compassion) among program completers.

### Positive Body Image, Body Functionality, and Embodiment: Conceptual and Empirical Support for Yoga-Based Interventions

Positive body image is a multi-dimensional concept that encompasses appreciation of the body's appearance and functionality, awareness of attentiveness to body experiences, and positive cognitions for coping with interpersonal challenges to a healthy body image (14). Body appreciation, a common quality of positive body image, accounts for unique variance in health outcomes and behaviors beyond that attributed to body dissatisfaction (15). Research has also shown that positive body image is a protective factor for negative body image; this finding is particularly relevant to the development of interventions (16, 17). Within the positive body image literature, research exploring *body functionality* (18–20), *functional body appreciation* (21), and *body appreciation* [e.g., (13, 14, 18, 22–25)] has flourished in recent years.

The embodiment model of positive body image is a related conceptual framework suggesting that engaging in embodying activities has the potential to cultivate the experience of positive body image through amplifying positive embodiment and reducing self-objectification processes (7, 26, 27). Five domains comprise *positive embodiment*: (a) connecting with one's own body in a positive manner that protects against negative bodily experiences; (b) living in the body with agency and appreciating its diverse functions; (c) engaging in self-care behaviors; (d) attending to and expressing the body's desires; and (e) considering the body a subjective, rather than objective, entity (27). Importantly, yoga has been hypothesized to improve overall embodiment by positively impacting each of these five dimensions (28). Further, the Attunement Model of Embodied Self-regulation and Well-being (AMESWR) conceptualizes yoga as a mindful self-care practice that supports positive embodiment (29, 30).

Yoga is an integrative physical and spiritual practice that aims to connect a person's mind, body, and spirit (31). Three core elements shared by the various forms of yoga include controlled breathing, meditative techniques, and physical postures (32, 33). Mindfulness is a key meditative component in traditional yoga classes such that yoga is often considered “mindfulness in movement” [(34), p. 28]. Originally derived from ancient Buddhist practices, mindfulness is the skill of being non-judgmentally aware of internal and external experiences in the current moment (34). The poses and breathing exercises often performed in yoga are intended to foster observing internal sensations rather than on monitoring appearance. Although aspects of yoga are considered a physical activity, it is grounded in philosophies that discourage competition and emphasize empowerment and community, which may increase its utility in fostering embodiment and positive body image effects.

For instance, Cox and Tylka's (35) conceptual model documents mechanisms through which yoga can support positive embodiment and embodying practices. Yoga provides state embodying experiences during its practice, including mindfulness, self-compassion, body appreciation, body image flexibility, perceived competence, joyful immersion, and connection to pleasure. These state embodying experiences then lead to more stable characteristics, resulting in stable embodying characteristics, though this transition is moderated by both individual and contextual variables.

Practitioners of yoga tend to focus on how the body feels internally rather than on how the body looks (36). Indeed, much like functionality-focused writing exercises (18), yoga practice was associated with lower self-objectification (4, 5, 7, 8, 37), higher health-related motivations for exercise engagement (37), higher levels of mindful eating (38), and aspects of embodiment (4, 7). Similarly, yoga practice was linked to ameliorating other eating disorder risk factors and to stimulating greater positive body image (e.g., body appreciation, body compassion, body gratitude) and self-care in youth and young adult samples (4, 8, 10, 39).

While interventions that deliver standard yoga over the course of several weeks have been effective for supporting positive embodiment, yoga also lends itself well to the incorporation of additional content explicitly focused on body image or mindfulness delivered in briefer formats. For example, Halliwell et al. (10) integrated components specifically focused on promoting positive body image, which primarily included the modification of the language used by the teacher throughout each of the classes during the 4-week yoga intervention. This brief intervention resulted in improved body appreciation, body satisfaction, positive mood, and body connectedness in the intervention as compared to the control group. In another study, a single yoga class was taught with three different approaches: a standard yoga class with neutral language, a mindfulness-based class and an appearance-focused class (40). While the appearance-focused class resulted in more body surveillance and less forecasted pleasure compared to the standard and mindfulness-based classes, the mindfulness-based class led to improved core affect and more remembered pleasure compared to the other two conditions.

### Yoga and Embodiment: What About Higher Weight Women?

The accumulating evidence base discussed thus far demonstrating the numerous benefits of yoga participation for aspects of body image and embodiment have almost exclusively been conducted in adolescent and young adult female samples that are not representative of the full spectrum of body weights and sizes (3, 4, 7, 8, 39, 41). Indeed, body size and weight stigma experienced by the yoga practitioner may moderate the association between the effects of yoga practice on positive embodiment, such that those with a larger body or with experience of weight stigma may not see the same level of effects of yoga practice on positive embodiment as (35). Research exploring the potential benefits of yoga on body image and embodiment among individuals of higher weight is limited, yet promising.

For example, participation in a 12-week yoga program reduced symptoms of binge eating and increased physical activity levels (42) among higher weight women. A related phenomenological qualitative study of women participating in the yoga program suggested the mechanisms by which these improvements occurred involved the program's encouragement of healthy reconnection to food and positive evaluation of physical well-being (43). More recently, Neumark-Sztainer et al. (44) qualitative analysis found that among their young adult participants (74% female), higher-weight status was more often associated with connecting a sense of achievement with the practice to positively impacting their experience of body image. Higher weight participants further tended to credit making upward social comparisons with others with negatively affecting body image to a greater degree than participants at lower body weights in the sample (44). Importantly, exposure to diverse bodies while practicing yoga was expressed as contributing to positively experiencing body image irrespective of weight status of the participants (44).

Women of higher weight may indeed benefit from participating in yoga; however, it would be remiss to encourage widespread uptake of the practice among this population without acknowledging the significant barriers they face in accessing yoga. Approximately 12% of people who have never tried yoga and 7% of lapsed yogis indicate that feeling that their “body type is not right for yoga” is a barrier to participation (45). Perhaps influencing these perceptions are dominant media misrepresentations of “the yoga body” within mainstream yoga-related print and social media, typically emphasizing women who appear thin and toned, young, White, and able-bodied (46–52).

Finally, women of higher weight and/or larger body size may have limited size-diverse yoga clothing and athletic wear options to choose from (53). Having access to trendy and functional clothing for exercise can promote physical activity and counter other barriers to exercise participation among women of higher weight, such as encountering and internalizing weight bias in exercise spaces (54), which may lead to body shame [i.e., negative, self-conscious response to a perceived failure to meet the standard body ideal; (55)], less motivation to do physical activity (56), and experiential avoidance [i.e., avoiding physical activity due to encountered or perceived stigma; (57)]. Despite that two-thirds of US women wear a size 14 or above (58), many yoga clothing and athletic wear brands do not offer sizes larger than 12. Brands that do offer extended sizing often carry these sizes exclusively online (e.g., Athleta), signaling to higher weight women that they may be unwelcome in brick-and-mortar stores, and further, within the broader yoga community (59).

Social justice-inspired researchers and activists have recently begun to explore how promoting diverse representations of “the yoga body” and increasing access to inclusive yoga spaces and leadership (e.g., instructors) may counter these barriers and contribute to advancing positive embodiment and health among higher weight women (60). Aligned with the Health At Every Size^®^ (HAES^®^) framework, which recognizes natural size and shape diversity in all bodies and encourages movement for enjoyment and enhanced quality of life (61), advocates for inclusive yoga have written extensively on the need for inclusion of people with diverse bodies in the yoga community [e.g., (60, 62–66)]. Relatedly, Pickett and Cunningham (67) recently interviewed leaders and instructors of body positive yoga classes and programs and found support for six strategies to develop and maintain inclusive yoga spaces: cultural commitment to diversity, language, authentic leadership, sense of community, leader advocacy, and a focus on health rather than weight. Similarly, Cook-Cottone and Douglass (68) offer additional actionable targets for enhancing diversity, inclusion, and accessibility within yoga spaces to deepen the cultivation of positive embodiment for all. One such avenue that may help amplify the reach of yoga to individuals of diverse and often marginalized social identities newer to the practice who may be reluctant to engage in in-person classes initially (e.g., possessing a larger body size) is through generating a greater online presence of yoga class offerings led by larger-bodied teachers (60).

### Efficacy of Brief, Web-Based, Guided Self-Help Interventions: Evidence From Mindfulness Self-Compassion Research

Given the significant barriers that women of higher weight may face when attempting to access yoga, web-based delivery methods may be a promising alternative. Moreover, given the ongoing COVID-19 pandemic and the cultural-shifts occurring around the acceptability of remotely-delivered programming, a web-based and scalable evidence-based intervention is increasingly relevant and critical. Even prior to the pandemic, web-based yoga programs have been found to be feasible and acceptable among various populations [e.g., (69, 70)]; however, little research has explored their effectiveness on improving body image and embodiment-related outcomes more specifically. Although there are many types of yoga practice, several yoga variations encompass mental health practices such as mindfulness (a key meditative component of yoga) and self-compassion [i.e., being non-judgmentally aware of experiences; (8)]. Thus, existing evidence supporting briefer, web-based delivery of mindfulness and self-compassion interventions to improve body image may provide preliminary support for web-based delivery of yoga programs with similar goals [e.g., (71)].

For instance, Albertson et al. (72) found that listening to guided self-compassion meditation podcasts had a positive impact on self-compassion and body image in women. Additionally, an online self-compassion writing intervention led to significant increases in self-compassion as compared to both traditional expressive writing and control groups (73). Similarly, web-based self-compassion-focused and body functionality-focused writing exercises led to higher body satisfaction and higher positive affect among college women (24). Another short, online body-focused gratitude (i.e. noticing and appreciating positive aspects of health, physical appearance, or body functionality) writing exercise resulted in lower weight bias internalization, greater body satisfaction, and more favorable appearance evaluation for those in the intervention compared to the control group (74). Finally, Toole and Craighead (75) found that brief exposure to self-compassion mediation training improved aspects of self-compassion and body image dissatisfaction in college women. Notably research to date has yet to evaluate the likely synergy between combining web-based size-inclusive yoga participation with a brief writing intervention that stimulates a focus on expressing gratitude, compassion, and appreciation for one's body functionality [e.g., (13, 23)].

Based on the amalgam of encouraging results of web-based mindfulness and self-compassion research [e.g., (71)], it is reasonable to hypothesize that a web-based yoga intervention may also promote aspects of positive embodiment. Web-based delivery may specifically benefit women of higher weight, who may experience physical activity-related discomfort and other noted barriers to yoga participation. Online yoga programs such as Curvy Yoga^®^, which is rooted in HAES^®^ principles and features body-diverse instructors, may empower participants with the opportunity to practice yoga in their own homes, at their own pace thereby countering many of the aforementioned barriers to participation (76).

### Specific Aims and Study Hypotheses

A brief, web-based, size-inclusive yoga program combined with daily body gratitude journaling may be a promising intervention for strengthening positive body image and other embodiment-related outcomes among women of higher weight who are new to yoga practice. Scalable, effective evidence-based body image interventions that are remotely delivered are particularly needed during the era of the COVID-19 pandemic and beyond. Extending previous lines of theory and research [e.g., (28, 35, 42–44), the present study aimed to evaluate the feasibility, acceptability and preliminary efficacy among completers (i.e., per-protocol analysis) of this integrative online mind-body program in a sample of ethnically-diverse college women of higher weight. We prioritized a per-protocol analysis for this early phase evaluation of efficacy given the anticipated moderately high attrition (71), that the project was not specifically powered to detect specific effects apriori, and given the more real-world considerations of self-selection for remaining engaged in a low-intensity, web-based self-help intervention (77). Therefore, outcomes related to preliminary efficacy are stated as exploratory research questions below. Based on the accruing evidence base previously reviewed, we predicted the following:

H1: We will observe moderately high attrition rates between 30 and 40% for the active size-inclusive yoga-body gratitude writing condition which is comparable to other brief, web-based mindfulness and self-compassion guided self-help interventions (71). Attrition for purposes of this analysis is calculated based on the number of participants who were randomized AND who completed the baseline data collection/orientation session but did not complete the post-intervention session.
H2: Women randomized to the active condition will endorse high levels of satisfaction (e.g., at least 75%) with the program.

Exploratory Research Questions: Will women randomized to the active condition demonstrate significant mean gains in aspects of positive body image, acceptance of physical activity discomfort, and self-compassion, as well as significant mean reductions in negative body image, weight bias internalization, and drive for leanness at the end of the 4-week protocol relative to baseline and the wait-list control condition?

## Materials and Methods

This study employed a randomized controlled trial (RCT) design. Data were collected before the COVID-19 pandemic from March 2016 through April 2017. This trial which was reviewed and approved under the old Common Rule is unregistered since it was completed prior to the January 2019 compliance date for the revised Common Rule and when the updated NIH definition of a clinical trial went into effect. Furthermore, this pilot study did not receive external funding from NIH. The data reported here were drawn from a larger parent study that included additional measures of eating behavior (e.g., intuitive eating, binge eating) and physiological functioning (e.g., blood pressure) that are not the focus of the current analysis.

### Participants

The study sample was comprised of 75 undergraduate cisgender females (*M*_Age_ = 21.2, *SD* = 2.5; *M*_BMI_ = 30.8 kg/m^2^, *SD* = 5.66) from a large publicly-funded institution located in the Southeastern United States. The sample was racially and ethnically diverse: 44% White/European American, 37.3% Black/African American, 6.7% Hispanic or Latina, 5.3% multiethnic/racial, 4% Asian/Asian American, 1.3% American Indian/Alaska Native, and 1% identity not listed. To be eligible to take part in the study, participants had to be an undergraduate female between the ages of 18–30, newer to the practice of yoga (i.e., practiced < once a week for the past 2 years), and have a minimum body mass index (BMI) of 25 kg/m^2^. The researchers used this imperfect metric as a proxy to represent size diversity given the goal to make this online wellness program more accessible and inclusive to individuals in larger bodies than had been in previous yoga-based research.

Prospective participants were found ineligible if they reported any of the following on the pre-screener: a regular yoga practice, a current eating disorder (e.g., self-reported bulimia nervosa), a physical disability that could limit their safe participation without additional guidance, had their physical activity currently monitored by a physician, were currently pregnant, had a seizure or neurological condition, had a pain condition that could impact their mobility and safe participation without more intensive guidance, had a heart condition, had high blood pressure, or had an implantable device (this was a safety precaution implemented due to the technology used to collect certain physiological data not reported here).

A total of 48 participants completed the study. Among the 42 (analyzed) completers with a *measured* baseline BMI of 25 or greater without substantial physiological missing data (21 females per condition), the mean age was 20.9 years (*SD* = 2.4) and the sample was predominantly White/European American (50%) and Black/African American (31%). Half of the completers consisted of advanced undergraduate students and 81% of the sample were non-psychology majors (see Table 1 for a summary). These percentages are similar to the campus population (78). The average BMI of the sample of analyzed completers was 30.9 (*SD* = 5.5).

**Table 1:** Demographic characteristics of participants at baseline (analyzed completers only).

**Variable**		
	***M***	***SD***
Age	20.98	2.38
Body mass index	30.99	5.49
	*N*	%
Race/ethnicity		
White/European American Black/African American Hispanic/Latino Other	21 13 3 4	50.0 31.0 7.1 9.6
Major		
Psychology Other	8 34	19.0 81.0
Academic rank		
Freshman Sophomore Junior Senior BeyondSenior	5 3 10 21 3	11.9 7.1 23.8 50.0 7.1

*N = 42; there were no significant (p < 0.05) between-group differences in demographic variables*.

### Measures

#### Demographic Questionnaire

This questionnaire asked participants their age, year in school, race/ethnicity, gender, and psychology major status.

#### Feedback Questionnaire

The participants in the experimental condition completed a feedback questionnaire that consisted of 12 quantitative items used to evaluate different qualities of feasibility (e.g., adherence), acceptability (e.g., satisfaction), and the participant's yoga practice environment. For example, participants in the yoga condition were asked questions about whether they completed the yoga practice with someone, how often they completed the yoga practices, how often they wore the motivational wristband, and where they typically practiced yoga. Participants also answered questions regarding their overall satisfaction with the program, how helpful it was to be provided yoga equipment, their sense of motivation from the daily images and wristband, and which yoga practice they found the most beneficial. This questionnaire also included 5 open-ended questions inviting participants to briefly share their perspectives on what barriers or challenges they faced, what benefits or successes they experienced, what aspect of the program they found most helpful, how the program could be improved in the future, and if they would recommend the program to a friend or family member.

#### Body Appreciation

The *Body Appreciation Scale-2* [BAS-2; (79)] was used to assess the degree to which one accepts and holds favorable opinions about one's body. An example item is “I appreciate the different and unique characteristics of my body.” This scale has 10 items that are rated on a five-point scale (1 = *never*, 5 = *always*). Scores are averaged to obtain an overall body appreciation score with higher scores reflecting higher body appreciation. This scale has demonstrated good internal consistency (Cronbach's alpha =0.97 for women) and construct validity as measured by expected associations to eating behaviors and body dissatisfaction (79). Cronbach's alpha was 0.91 at both baseline and post in the present study.

#### Body Image Flexibility

The 12-item *Body Image-Acceptance and Action Questionnaire* [BI-AAQ; (80)] was administered to examine participants' body image flexibility. Sandoz et al. (80) define body image flexibility as “the capacity to experience the ongoing perceptions, sensations, feelings, thoughts, and beliefs associated with one's body fully and intentionally while pursuing chosen values” (p. 3). Higher scores indicate greater body image flexibility in the original validation sample (80). Cronbach's alphas suggest excellent internal consistency (α = 0.92). As expected, the measure correlated negatively with body image dissatisfaction, and it predicted disordered eating even after controlling for BMI and body dissatisfaction. Test-retest reliability analysis yielded good stability over a time frame of 2 to 3 weeks (α = 0.80) Cronbach's alpha was 0.90 at baseline and 0.92 at post in the present study.

#### Functional Body Satisfaction

The Functional Satisfaction subscale of the *Embodied Image Scale* (81) was used to assess participants' feelings towards what their body can do. An example item includes: “I feel really good about what I can do physically.” This subscale includes three statements that are rated on a five-point scale (1 = *not at all true of me*, 5 = *very true of me*); higher scores indicate greater functional body satisfaction. This measure's convergent validity was demonstrated via expected associations with self-esteem (81). This subscale yielded internally consistent scores with adults [Cronbach alpha =0.91; (82)]. Cronbach's alpha was 0.85 at baseline and 0.87 at post in the present study.

#### Functional Body Awareness and Appreciation

The *Functional Body Awareness and Appreciation Scale* [FBApS; (21)] was used to examine body functionality awareness and body functionality appreciation. This is an 11-item measure that is rated on a five-point scale (1 = *Strongly Disagree*, 5 = *Strongly Agree*). This measure consists of two subscales: functional body appreciation and functional body awareness. The functional body awareness subscale measures how connected one is to their body (e.g., “I have paid attention to the changing sensations of my body”). The functional body appreciation subscale assesses how much one values the physical functioning of their body (e.g.,“I have been grateful for what my body has allowed me to do”). Higher scores indicate greater awareness of body functioning or appreciation of body functioning. Cronbach's alpha was 0.83 for the awareness subscale and 0.82 for the appreciation subscale in the original validation sample (21). This measure demonstrated convergent validity through expected associations body surveillance and depressive symptoms (21). Cronbach's alpha for the awareness subscale was 0.81 at baseline and 0.80 at post in the present study. The internal consistency of the appreciation subscale was 0.60 at baseline and 0.72 at post in the current sample.

#### Body Image Shame

Body shame was measured using the *Body Image Shame Scale* [BISS; (83)]. This scale consists of 14 items that are rated on a five-point scale (1 = *Never*, 5 = *Almost Always*); higher scores indicate more body image shame. This measure includes two subscales: an externalized dimension of body shame (BISS-External) and an internalized dimension of body shame (BISS-Internal). The BISS-External subscale measures one's belief that society is negatively evaluative of one's body. An example item is “I feel uncomfortable in social situations because I feel that people may criticize me because of my body shape.” In contrast, the BISS-Internal subscale measures one's own negative evaluations of their body and subsequent concealment efforts. An example item is “I choose clothes that hide parts of my body that I consider ugly or disproportional.” Evidence for this measure's convergent validity includes expected associations with eating pathology, depression, and physical appearance comparison (83). Robust internal consistency reliability estimates were observed for both the BISS-External (α = 0.89) and BISS-Internal (α = 0.90) in the original validation study conducted with Portuguese women (83). The BISS-Internal subscale also yielded internally consistent scores in an American undergraduate female sample [Cronbach's alpha = 0.93; (84)]. Cronbach's alpha was 0.87 at baseline and 0.90 at post for the BISS-Internal subscale and was 0.90 at baseline and post for the BISS-External subscale in the present study.

#### Weight Bias Internalization

Internalized weight bias was assessed using the original *Weight Bias Internalization Scale* [WBIS; (85)]. This scale measures the degree to which people accept negative weight-related stereotypes and apply these stereotypes to themselves. An example item includes: “Because I'm overweight, I don't feel like my true self.” This measure consists of 11-items that are rated on a seven-point scale (1 = *strongly disagree*, 7 = *strongly agree*). Items are summed and then averaged to produce a total score with higher scores suggesting stronger internalized weight bias. This scale yielded internally consistent scores (Cronbach's alpha = 0.90) and it demonstrated convergent validity as measured by predicted associations to anti-fat attitudes, body image, and disordered eating (85). Cronbach's alpha was 0.89 at baseline and 0.88 at post in the present study.

#### Drive for Leanness

Drive for leanness was measured with the *Drive for Leanness Scale* [DLS; (86)]. This scale examines participants' desire to have relatively low body fat and toned muscles. This measure consists of six items that are rated on a six-point scale (1 = *never*, 6 = *always*). Items are summed to create a total score with higher scores indicating a greater desire to achieve a lean body figure. One item on the scale is “My goal is to have well-toned muscles.” This scale demonstrated convergent validity through its associations with drive for thinness and drive for muscularity and it added discriminant validity to existing body image measures (86). Previous work with this scale has demonstrated good internal consistency for women [Cronbach's alpha =0.84; (87)]. Cronbach's alpha was 0.85 at baseline and 0.80 at post in the present study.

#### Physical Activity Acceptance

Physical activity acceptance was examined with the *Physical Activity Acceptance Questionnaire* [PAAQ; (88)]. This scale assesses participants' ability to accept the normative discomfort that may accompany exercising and physical activity. This measure has 10 items that are rated on a seven-point scale (1 = *never true*, 7 = *always true*). This scale has two subscales: Cognitive Acceptance and Behavioral Commitment. The Cognitive Acceptance subscale measures participants' willingness to engage in physical activity even while they are having discouraging thoughts. An example item is: “If I have the thought “exercising today won't be enjoyable,” it derails me from my exercise plan.” The Behavioral Commitment subscale measures one's ability to continue exercising despite difficult feelings or sensations. An example item is: “Even if I have the desire to stop while I am exercising, I can still follow my exercise plan.” This scale demonstrated good internal consistency for the overall score (Cronbach's alpha = 0.89) and the subscales had alpha levels of 0.83 (Cognitive Acceptance) and 0.85 [Behavioral Commitment; (88)]. This measure has shown predictive validity for objectively assessed physical activity (88). Cronbach's alpha was 0.86 at baseline and 0.83 at post for the Cognitive Acceptance subscale and was 0.74 at baseline and 0.82 at post for the Behavioral Commitment subscale in the present study.

#### Self-Compassion

The 26-item *Self-Compassion Scale* [SCS; (89)] was used to measure participants' self-compassion as conceptualized by Neff (90). Items belong to one of three lower-level constructs: self-kindness (vs. self-judgment), common humanity (vs. isolation), and mindfulness (vs. over-identification). Collectively, they form a higher-order construct of self-compassion. An example item includes “When things are going badly for me, I see the difficulties as part of life that everyone goes through.” Using a scale from 1 (almost never) to 5 (almost always), higher scores (subscale and total) indicate greater self-compassion. Cronbach's alphas suggest excellent internal consistency for all three domains as well as higher-order factor (α = 0.92). As expected, the scale is inversely correlated with measures of self-criticism, depression, and anxiety (89). Test-retest reliability analysis yielded excellent stability of the SCS scores over a 3-week period (α = 0.92). Cronbach's alpha was 0.91 at baseline and 0.92 at post in the present study.

### Procedure

Following IRB ethics approval (#16-01-01), participants were recruited through the Department of Psychology's online research sign-up system to participate in a 4-week mind-body health promotion program for women with a BMI of 25 or greater. Participants completed an online pre-screener and were subsequently invited to attend the baseline in-person session if eligible where they provided written informed consent. Eligible participants were then randomized using a random-number generator prior to coming in for the baseline data collection and orientation sessions. Participants completed a confidential Doodle poll to facilitate scheduling these sessions. Orientation/data collection sessions were held in groups of two to seven participants. Across both conditions (experimental and wait-list), participants completed the set of paper and pencil measures sequenced in the same order.

To enhance motivation and minimize a potential barrier to engaging in the yoga practices, participants in the experimental condition received a yoga equipment kit (i.e., block, strap, and mat) to use during the 4-week intervention. To further incentivize returning for the post-program data collection session, mild deception was conveyed to participants randomized to the yoga and body gratitude writing condition. They were told that they would need to return the yoga kit at the follow-up visit when in fact these kits were theirs to keep. As another attempt to enhance engagement, the yoga condition participants were also given and encouraged to wear a motivational wristband stating “Yoga is for EVERY Body! No weigh-in Necessary!”.

Each week, participants in the experimental group were asked to practice yoga at least 3 times per week, using a 15-min Curvy Yoga Studio^®^ Practice YouTube Video link sent to them at the beginning of each week *via* email. Anna Guest-Jelley was the instructor who led all of the yoga video practice sequences. Anna Guest-Jelley is a larger-bodied, White female yoga professional and yoga inclusion and body acceptance advocate. She is the founder of Curvy Yoga^®^ (www.curvyyoga.com) instituted in 2010 which is currently a leading online resource for accessible body positive and body-affirming yoga regardless of one's size or ability. She is also the author of *CurvyYoga*^®^: *Love Yourself & Your Body a Little More Each Day* (76) and the co-editor of and contributor to *Yoga and Body Image: 25 Personal Stories about Beauty, Bravery & Loving Your Body* (63).

In week 1, participants were invited to engage with the “Morning Wake-up” practice. This practice provided a gentle yet energizing sequence that included breath awareness, reclining and seated twists, partial Sun Salutation (Surya Namaskaram) with forward fold (Uttanasana), high lunge (Anjaneyasana) and downward facing dog (Adho Mukha Svanasana) offered as an option. The sequence concluded with extended side angle (Utthita Parsvakonasana) alternating with tree pose (Vrksasana) and culminating with the “Breath of Joy” awakening pranayama breathing practice.

In week 2's “Find Your Steady and Sweet” video practice, Anna sought to inspire participants to find that balance between effort and ease which is a foundational yogic principle grounded in the *Yoga Sutras of Patanjali* (91). This video in addition to offering a gentle flow of standing poses [e.g., high lunge into pyramid (Parsvottanasana), downward facing dog, warrior II (Virabhadrasana II), extended side angle, wide-legged forward fold (Prasarita Padottanasana)], the sequence also integrated in a lovingkindness practice to encourage body acceptance linked to movement and breath. The phrases included: “May I greet my body with gentleness”; “May I soften when life invites me to harden”; “May I listen to my intuition with wisdom and trust it with ease”; “May I appreciate my body a little more in this moment just as it is.”

In week 3's “Living Your Body Positive Intention” video practice, participants were encouraged to engage in another mostly standing pose flow that built on the other practices by incorporating triangle pose (Trikonasana) and chair pose (Utkatasana). A series of lovingkindness affirmations were offered to link breath to the beginning of the partial Sun Salutation sequence: “I am grounded.”, “I am loved.”, “I am enough.”, “I am peace.”, and “I am whole.”. This practice concluded with a brief seated silent meditation. In week 4, participants were invited to “choose their own adventure” by selecting from any of the previous three videos to use for their yoga practice. Across all video practices, setting an intention, using supportive props (e.g., blocks, blanket, and the wall) alongside going at one's own pace were encouraged.

In concert with the web-delivered yoga practice videos, participants were asked to complete a Curvy Yoga^®^ daily body gratitude journal prompting them to write down one aspect they were grateful that their body enabled them to do that day. This was asked of participants to complete regardless of whether they practiced yoga that day or not. Participants received four copies of the body gratitude journal template sheets during their initial study visit as well as a digital version of the template sent via email in conjunction with their first week's daily yoga practice link. To avoid any concern of evaluation, participants were informed that their body gratitude journals would not be collected at the conclusion of the study and are instead an opportunity to reflect on their experience independently. The single, unstructured body gratitude journaling prompt was adapted from the work of Dr. Jessica Alleva and colleagues' Expand Your Horizon program's more structured writing prompts [e.g., (18)]. These prompts led to improvements in body satisfaction among undergraduate women (18).

Finally, to strengthen engagement given the low-intensity of direct contact with the research team, participants were emailed a daily motivational image, featuring quotes reflecting themes of positive embodiment and body inclusivity in yoga (e.g., “There is no wrong way to have a body.”; “Yoga is not about the shape of your body. It's about the shape of your life.”; “I intend to accept my body today, love my body tomorrow & appreciate my body always.”; “The attitude of gratitude is the highest yoga.”; “I will treat my body with respect and kindness. I will feed it, keep it active and listen to its needs. I will remember that my body is the vehicle that will carry me to my dreams”).

All program completers attended a second in-person session following the same procedure as described above. At the end, participants in the yoga condition were invited to provide written feedback and offered to keep their yoga equipment kit. At the conclusion of the study, participants randomized to the wait-list condition were granted access to the videos and motivational images used during the intervention. As an additional incentive, all participants received a $10 (baseline) and a $20 (follow-up) Amazon gift card.

## Results

### Preliminary Analysis

**Figure 1:**
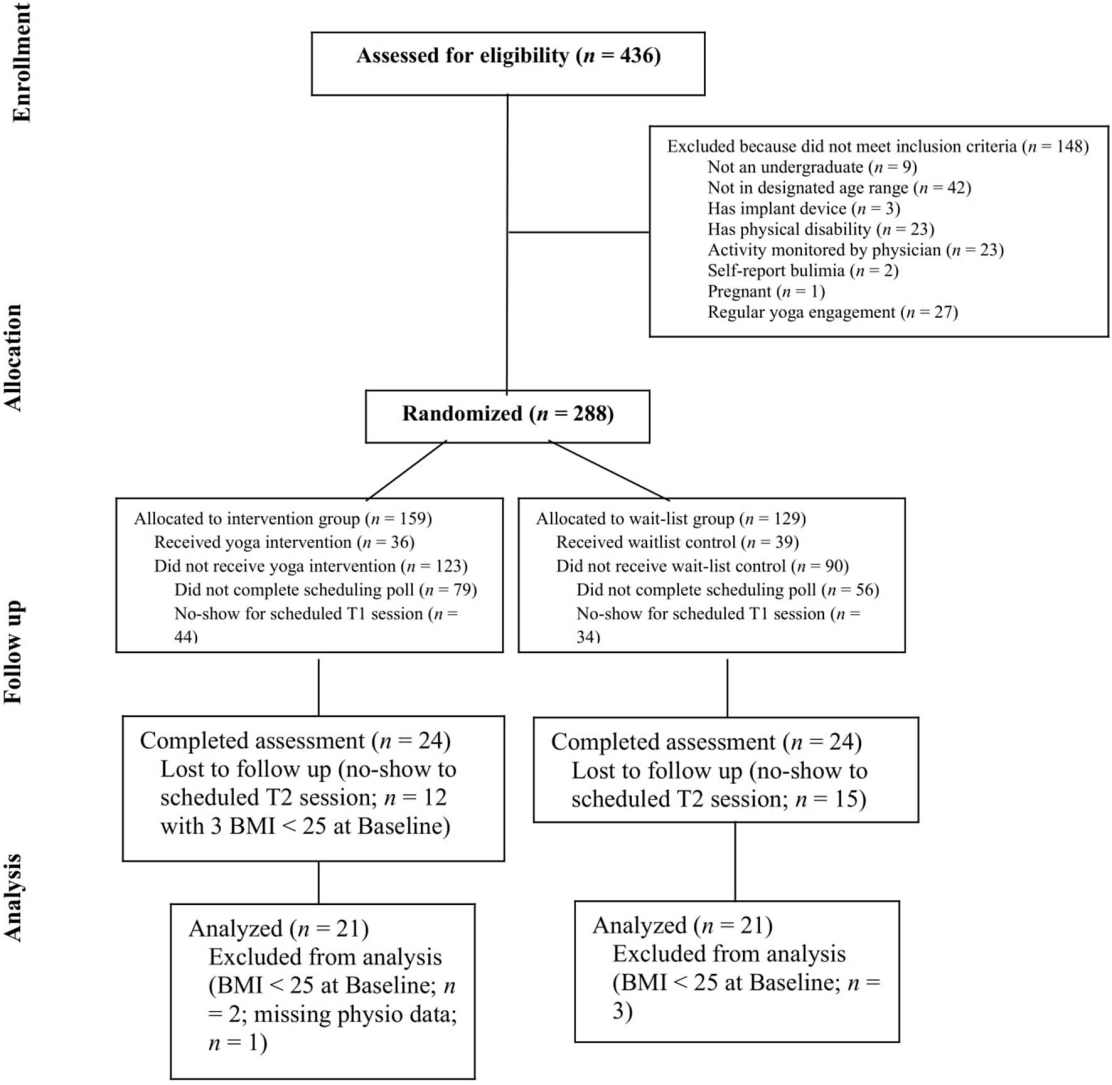
CONSORT flow diagram.

The CONSORT flow diagram (see Figure 1) specifies participant randomization and attrition over the course of the study. Initially, 288 eligible participants were randomized to the experimental yoga and body gratitude writing condition (*n* = 159) or to the wait-list control condition (*n* = 129). During the initial randomization period, 172 participants did not receive the condition they were allocated to primarily for failing to schedule or attend the baseline data collection and orientation sessions. This resulted in 36 yoga condition participants and 39 wait-list control participants completing baseline measures. Twenty-seven participants were lost to follow-up and did not complete post-test measures. In adhering to our per-protocol analysis inclusion criteria, an additional 5 participants with measured BMI <25 were excluded from the reported analyses. A final participant's data were not included in the analyses due to a missing BMI data point at baseline as a function of the constraints of the measurement device used. This resulted in an analyzed baseline sample of 66 participants (30 in the yoga condition and 36 in the wait-list control condition) and 42 participants who were analyzed as completers (21 per condition).

All analyses were calculated using IBM SPSS statistical software. Data were initially screened to ensure they met assumptions for parametric statistical procedures including analysis of variance (ANOVA) and paired *t-*tests. Among the 42 analyzed completers, the amount of missing data at the item-level was negligible across all variables (<0.02% at both baseline and post). Therefore, the mean substitution strategy was used (92). In addition to inspecting the distributional characteristics of variables with histograms, boxplots, skewness, and kurtosis, the Shapiro-Wilk test was used to assess for normality. This approach is recommended over the Kolmogorov-Smirov test given the sample size of completers for our per protocol analysis was <50 (93). At baseline, all of the Shapiro-Wilk tests were *p* > 0.05. At post, the Shapiro-Wilk test was *p* < 0.05 for the body functionality satisfaction variable. However, the skewness and kurtosis metrics were within acceptable limits (94) and therefore, no additional data transformations were implemented. Homogeneity of variance was confirmed across all variables at baseline and post (Levene's tests *ps* > 0.05). There were no baseline differences between conditions on the variables of interest indicative of a successful randomization procedure (all *ps* > 0.05).

### Feasibility and Acceptability

Over the course of the study recruitment period, significant interest was stimulated by the project as evidenced by 436 individuals completing the initial prescreener. Nearly 300 individuals were found eligible after completing the prescreener yielding a 66% eligible recruitment rate. The overall study attrition rate of 36% was within the expected range for brief, low-intensity web-delivered guided self-help mindfulness and self-compassion-based programs (71). Please note that attrition rates are based on participants who were randomized AND who completed the first baseline data collection/orientation session but did not complete the post-intervention session. Unfortunately, as noted earlier, the randomization process we enacted by randomizing at enrollment rather than at the baseline sessions artificially inflated “drop-out” due to the high number of no shows to the baseline sessions once individuals were found eligible and enrolled remotely.

The attrition rate for the yoga and body gratitude writing condition (i.e., 33%) was slightly lower than the corresponding rate observed among the wait-list control (i.e., 38%). Among the participants in the active experimental condition, measures of self-reported adherence revealed that 81% practiced with the yoga videos at least 3–4 times per week, 71% wrote in the body gratitude journal at least 1–2 times per week, and 52% wore their motivational wristband at least 1–2 times per week. Notably, 29% of participants reported not writing in their journals at all throughout the program. In terms of program acceptability, 86% of participants were at least moderately satisfied with the program overall. Seventy-six percent of participants also found the daily images at least somewhat motivating or encouraging and all participants reported having the yoga equipment kit supplied to them at least somewhat helpful. The following percentages reflect which of the three online yoga practice videos participants found most beneficial during the 4-week mind-body wellness promotion program: “Morning Wake-up”: 28.6%, “Find Your Steady and Sweet”: 14.3%, “Living Your Body Positive Intention”: 23.8%, None: 4.8%, and All: 4.8%. Please see Table 2 for additional details characterizing aspects of the participants' experience with the program.

**Table 2:** Participant responses to feedback questionnaire (*N* = 21).

**Question**	**Response Frequencies**				
	**Not at All**	**Slightly**	**Somewhat**	**Moderately**	**Very**
Overall, how satisfied are you with the size-inclusive mind-body health promotion Yoga program you participated in over the past 4 weeks?	0	1	2	8	10
How motivating or encouraging did you find the daily body acceptance images and words emailed to you?	0	5	5	3	8
How helpful was it to be able to have the Yoga equipment provided by our research team for you to use?	0	0	2	3	16
If you did wear the motivational wristband at some point during the 4-week study how motivating or encouraging did you find wearing it?	0	2	3	3	3
	0	1–2	3–4	5–6	7
How many days per week did you complete the 15-min Yoga practice?	0	4	7	9	1
How often per week (in days) did you write in your daily body gratitude journal?	6	7	4	2	2
How often per week (in days) did you wear the motivational wristband?	10	3	2	1	5
	0	1	2	3+	
How many times per day did you complete the 15-min Yoga practice?	0	19	2	0	
Which of the three 15-min Yoga practices did you find most beneficial?	Morning wake-up	Find your steady and sweet	Living your body positive intention	None	All
	6	3	5	1	1
Did you tend to practice the Yoga sessions alone or with others most of the time?	All alone	With others	Both equal		
	19	0	2		
	Female roommate or friend	Male roommate or friend	Romantic partner	Female family member	Male family member
If you practiced with someone else at some point during the 4-week program who did you practice with most of the time?	1	1	4	5	5
	Home	Friend's house	Romantic partner's house	Family member's house	Other
Where did you tend to practice the Yoga sessions most of the time?	17	1	2	1	0

Qualitative analysis of identifying patterns in the responses to the five open-ended questions was guided by Saldaña's (95) hypothesis coding approach and conducted by the first author. A priori codes were identified based on the anticipated content of each question posed to participants in the feedback questionnaire. Given the space limitations, we prioritized reporting the number of participants endorsing a particular theme rather than also including exemplar quotes.

The primary barrier experienced (which was expressed by 12 participants) was the challenge of managing time and incorporating the practices in their daily routine as a busy college student. Difficulty maintaining sufficient motivation for engaging in the program was reported by 3 participants. Meanwhile another 3 participants reported physical limitations (e.g., limited lower-body flexibility, poor balance) or illness as a barrier to their experience. Participants expressed several benefits and successes experienced during the program. These included increased appreciation or acceptance of their body or weight (5 participants), strengthened sense of calm and relaxation (6 participants), enhanced appreciation of aspects of body functionality (4 participants), improvements in mood, enjoyment, and happiness (3 participants), improved self-worth or positive self-evaluation (3 participants). Two participants also appreciated how the program promoted a more consistent exercise routine. Meanwhile a minority of others expressed noting improvements in sleep, mental alertness, energy, and pain relief.

Similarly, participants acknowledged a number of helpful aspects of the program. Five participants particularly highlighted the size-affirming quality of the program including having an instructor living in a larger body. Participants further found the motivational elements of the program helpful including the specific body positive images and quotes (3 participants), the overarching message of empowering loving one's body and coping with life stress (4 participants), and making more time to be physically active (2 participants). Two participants found the briefer nature of the practices most helpful, two listed the active yoga practice as most useful, and another two appreciated that the equipment was supplied beforehand, which facilitated their engagement in the program. Others recognized the journaling, setting a daily intention, practicing the lovingkindness mantras, realizing the importance of self-care, or increasing awareness of body functionality as particularly helpful in their experience of the program.

In conjunction with the largely beneficial experiences reported, participants offered a few areas for improvement. Five participants provided suggestions that reflected ways of enhancing the delivery format to minimize technical difficulties and streamline access to content (e.g., using a webpage that links video material or include videos in daily emails with the motivational images; 3 participants) or to capitalize on mobile technology (e.g., having the journal be accessed online through an app with body positive messages; 2 participants). Four participants would have preferred increased variety and/or intensity of the yoga practices. Meanwhile, three participants expressed an interest in incorporating more contact with other participants and a yoga teacher through live group classes. Others offered recommendations to remind participants that for the first 3 weeks there was only one yoga video practice, to record practices as a way to promote safety, or to offer a weekly check-in with the research team. One participant would not want the journaling component as an expectation in a subsequent revision of the program.

Lastly, all but one of the 21 participants in the yoga and body gratitude writing condition would recommend the program to a friend or a family member. The reasons why the large majority would recommend the program echo the previously mentioned benefits. These included the size-affirming nature of the program which challenged stereotypes of who can do yoga (6 participants), how the program encouraged body acceptance (3 participants) and included messages encouraging positive self-worth or feelings about the self (4 participants). Others would suggest the program to help people in their life find peace of mind or improve their mood (2 participants), to encourage lifestyle change (2 participants), because they found the experience fun and enjoyable (2 participants), or helped motivate themselves to want to do more yoga (2 participants). A few participants further expressed that people they know would appreciate the beginner level of the practices, the brief time commitment, and the stress relieving and relaxation enhancing elements of the program. The single participant who would not recommend the program indicated that they did not like the emphasis on size acceptance at higher body weights. They believed that it was not okay to be “fat” or “overweight” even if one could practice yoga. This sentiment clearly reflects the tenacity of internalized weight stigma perpetuated by the pervasiveness of diet culture.

### Per-Protocol Preliminary Efficacy

A series of 2 (Condition: yoga vs. wait-list) x 2 (Time: baseline vs. post) mixed design repeated measures ANOVA models were computed to provide a preliminary evaluation of potential intervention effects on the outcomes of interest. Cohen's *f* were calculated to evaluate the effect size estimates for omnibus Time main effects and Condition x Time interaction effects (96) and interpreted as reflecting small (0.10), medium (0.25), and large (0.40) magnitudes (97). Effect size estimates for significant baseline-post mean differences in follow-up analyses were computed using Cohen's *d* (96) and were interpreted as indicating small (0.20), medium (0.50), and large (0.80) magnitudes (97).

Across groups, several medium to large-sized main effects of Time emerged. More specifically, over the 4-week intervention, significant declines in external body shame, [F (1, 40) = 5.27, *p* = 0.03, *f* = 0.32, (95% CI: 0, 0.66), observed power = 0.61], internal body shame, [F (1, 40) = 5.71, *p* = 0.02, *f* = 0.33, (95% CI: 0.03, 0.68), observed power = 0.65], and weight bias internalization, [F (1, 40) = 5.88, *p* = 0.02, *f* = 0.34, (95% CI: 0.04, 0.68), observed power = 0.66] were observed. Similarly, over time significant improvements were shown for body image flexibility [F (1, 40) = 5.15, *p* = 0.03, *f* = 0.31, (95% CI: 0, 0.66), observed power = 0.60], body appreciation [F (1, 40) = 13.22, *p* < 0.001, *f* = 0.54, (95% CI: 0.23, 0.88), observed power = 0.94], functional body appreciation [F (1, 40) = 5.07, *p* = 0.03, *f* = 0.31, (95% CI: 0, 0.66), observed power = 0.59], functional body awareness [F (1, 40) = 6.18, *p* = 0.02, *f* = 0.35, (95% CI: 0.06, 0.69), observed power = 0.68], and self-compassion [F (1, 40) = 5.45, *p* = 0.03, *f* = 0.33, (95% CI: 0.01, 0.67), observed power = 0.63].

Additionally, as shown in Table 3, analyses produced significant medium to large-sized interaction effects of Condition x Time to further clarify several of the main effects of Time described above. These included functional body satisfaction (observed power = 0.69), internal body shame (observed power = 0.81), physical activity acceptance-behavioral commitment (observed power = 0.59), body appreciation (observed power = 0.63), functional body appreciation (observed power = 0.56), and functional body awareness (observed power = 0.74). Follow-up paired *t*-tests (*ps* all < 0.05) revealed that the observed gains in multiple facets of positive body image and behavioral commitment to engaging in physical activity (*ds*: −0.52 to −0.75) and reduction in internal body shame (*d* = −0.73) experienced by the participants randomized to the yoga and body gratitude writing condition ranged from medium to large in magnitude. All *p*-values were >0.05 when examining the baseline-post change in means for the wait-list control condition.

**Table 3:** Means and summary statistics for mixed-model analysis for outcome variables for yoga condition by assessment time from pre- to posttest.

	**Pretest**		**Posttest**		**Condition by time**			
	**Yoga**	**Control**	**Yoga**	**Control**				
	**(*n* = 21)**	**(*n* = 21)**	**(*n* = 21)**	**(*n* = 21)**				
**Variable**	***M (SD)***	***M (SD)***	***M (SD)***	***M (SD)***	***F***	***p***	***f***	**95% CI**
Functional body satisfaction	2.59 (0.96)	2.90 (1.12)	3.17 (0.95)	2.79 (0.95)	6.35	0.016	0.36	0.07–0.70
Internal body shame	2.23 (0.89)	2.15 (0.77)	1.67 (0.84)	2.20 (1.00)	8.41	0.006	0.42	0.13–0.76
External body shame	2.18 (0.97)	1.66 (1.17)	1.79 (0.99)	1.63 (1.20)	3.71	0.061	0.25	0–0.60
Weight bias internalization	3.79 (1.24)	3.57 (1.14)	3.18 (1.07)	3.46 (1.28)	2.76	0.104	0.20	0–0.56
Physical activity acceptance (cognitive acceptance)	19.09 (6.86)	16.15 (7.47)	17.76 (6.46)	16.38 (7.40)	1.23	0.274	0.07	0–0.47
Physical activity acceptance (behavioral commitment)	16.95 (5.88)	18.24 (5.98)	19.42 (6.78)	17.90 (5.49)	5.01	0.031	0.31	0–0.66
Drive for leanness	3.22 (1.11)	3.60 (1.05)	2.98 (1.07)	3.56 (0.86)	1.73	0.096	0.13	0–0.51
Body appreciation	3.22 (0.88)	3.33 (0.79)	3.72 (0.62)	3.44 (0.89)	5.47	0.024	0.33	0.01–0.67
Functional body appreciation	3.03 (0.64)	3.17 (0.59)	3.50 (0.67)	3.18 (0.81)	4.67	0.037	0.30	0–0.64
Functional body awareness	3.24 (0.77)	3.68 (0.65)	3.70 (0.59)	3.67 (0.82)	7.09	0.011	0.38	0.09–0.72
Body image flexibility	47.14 (12.58)	51.00 (13.64)	53.38 (13.82)	52.90 (17.22)	1.46	0.234	0.10	0–0.49
Self-compassion	2.83 (0.57)	2.79 (0.54)	3.11 (0.61)	2.84 (0.61)	2.18	0.147	0.17	0–0.53

### Baseline Differences Between Completers vs. Non-completers

The present early-stage RCT prioritized the per-protocol analysis to explore the presence of preliminary estimates of intervention effects under the most optimal conditions. Yet this approach may reflect greater ecological validity for targeted individuals who sustain engagement in a minimally-guided web-based self-help program (77). Given that estimates may be biased in not accounting for attrition, we conducted a series of *post-hoc* ANOVA models in order to evaluate whether participants who completed the post-test measures at the conclusion of the 4-week intervention and were included in the analyses (*n* = 42) differed on the variables of interest at baseline from participants who dropped out (*n* = 24). Analyses revealed that at baseline, completers reported higher levels of functional body satisfaction (*p* < 0.001), body image flexibility (*p* < 0.001) alongside lower levels of weight bias internalization (*p* < 0.05), and drive for leanness (*p* < 0.05) relative to non-completers. There were no between group baseline differences observed for age, BMI, body appreciation, functional body appreciation, functional body awareness, physical activity cognitive acceptance and behavioral commitment, internal body shame, external body shame, and self-compassion (*p-*values all > 0.05). A chi-square analysis further confirmed that the groups also did not differ with respect to the representation of race and ethnicity at entry (*p* > 0.05).

## Discussion

Aligned with relevant embodiment theory (26, 28–30), the present pilot RCT was designed to evaluate the feasibility and acceptability and to explore the preliminary efficacy of a 4-week online yoga and body gratitude journaling intervention for improving aspects of positive body image, self-compassion, and physical activity acceptance, and for reducing negative body image, weight bias internalization, and the drive for leanness among higher weight college women. In doing so, our research was responsive to recent calls to advance greater diversity, equity, inclusion, and access to yoga-based wellness and positive embodiment science and practice (44, 60, 67, 68). This initial stage analysis also specifically serves to address critical gaps in dissemination science seeking to develop and test the effectiveness of scalable, remotely-delivered guided self-help interventions to bolster college student mental health especially in the ongoing modern post-COVID-19 era (98).

We found strong support for the feasibility of the program as indexed by the high student interest translating into a moderately-high eligibility rate coupled with a reasonable attrition rate that fell within the expected range for brief, low-intensity, online mindfulness-based interventions (71). Given the intention to model a minimally-guided self-help approach, we adopted a more conservative set of eligibility criteria than may be the case when there is greater planned engagement with members of the research team. At the same time, in anticipation of the need to compensate for limited research team interactions, we built in additional intervention components to stimulate retention. These strategies included sending daily motivational images and encouraging participants to wear a motivational wristband with the former being more appealing than the latter for such purposes. Meanwhile, high program satisfaction was observed along with high self-reported adherence for using the yoga practice videos with more modest corresponding adherence for completing the body gratitude journaling. Notably a significant minority of participants reported not completing the journaling component and almost half did not wear the motivational wristband as intended as part of the program. Qualitative feedback provided additional support for the range of benefits experienced aligned with the preliminary quantitative findings while also pointing to important considerations for improving the design of the program in the future.

Further, despite being under powered in some cases, results demonstrated participants in the yoga and body gratitude journaling condition experienced significant moderate to large-sized gains in several dimensions of positive body image (including components of body functionality) and behavioral commitment to physical activity alongside a meaningful decline in internal body shame relative to a wait-list control over the 4-week intervention period. These preliminary findings are consistent with contemporary conceptual models of yoga and positive embodiment [e.g., (28, 35)] and support the accruing evidence base of yoga, self-compassion, body functionality/gratitude writing, and body image research conducted in western (young adult) female samples [e.g., (3, 4, 8–10, 13, 23, 72, 75, 77, 99–101). Below we outline the specific ways in which our study uniquely contributes to furthering innovations in body image intervention science for women.

First, limited yoga intervention research has specifically targeted the inclusion of higher weight college women who may feel stigmatized and alienated in practicing yoga in conventional mainstream wellness spaces (44, 60). Our study expands upon the work of McIver and colleagues from a more weight-neutral perspective that does not center eating pathology and weight management as a major emphasis (42). Notably, in support of their earlier efforts, we found that our brief online intervention yielded benefits for strengthening participants' orientation towards physical activity more broadly and in particular a more positive and empowered connection to one's experience of the physical body (43). Therefore, yoga may be a promising holistic approach to cultivating the willingness among individuals living in larger bodies to persist in meaningful physical activity amidst the normative discomfort that may arise in the process (88). This perspective aligns with classic yoga philosophy that encourages practitioners to find their own particular balance between effort and ease within the postures (91). It is also possible that another unique element of the present study by featuring a larger-bodied yoga teacher as a model of positive embodiment for the online practices conveying accessible, choice-driven language may have further reinforced these experiential qualities for participants as counter to less affirming media representations of yoga embodiment (44, 46–48, 60, 67). Nevertheless, it is likely that a more intensive and explicit approach may be warranted to influence substantive changes in the more cognitive dimension of physical activity acceptance.

Second, to our knowledge, the current study is the first online yoga intervention that specifically centers the experiences of body image and embodiment in a relatively brief, month-long timeframe that offered shorter practice lengths (~15 min) than previous comparable scholarship [e.g., (3, 4, 9)]. One notable exception was Halliwell and colleagues' (10) study that exposed participants to a 4-week, 4-session yoga intervention for improving positive body image, embodiment, and mood in college women. However, the program still relied on weekly, in-person 60-min yoga classes. As people around the globe continue to navigate the adversities of the persistent COVID-19 pandemic, it is imperative that behavioral health and wellness promotion programs (inclusive of those targeting body image processes) are able to remain accessible through virtual and other remotely-delivered options. Additionally, the emphasis on providing opportunities to engage with shorter practices may have sustained motivation especially for participants who were busy college students newer to yoga and not participating for academic course credit, as an alternative to a required school-based physical education curriculum [e.g., (8)] or as an adjunctive therapy to an outpatient eating disorder treatment program [e.g., (9)]. This particular design feature also lended itself well to the low-intensity, more self-guided format implemented.

Third, our research both complements and extends previous intervention programs which investigated the potential benefits to body image after exposure to yoga infused with explicit themes and/or language encouraging compassion, awareness and appreciation of body functionality/internal experience, and other aspects reflective of positive embodiment [e.g., (4, 8–10)]. Indeed, our pilot RCT is the first of its kind to innovatively combine this approach to amplifying the existing embodying power of yoga with a body gratitude writing component. Including this latter strategy was inspired by the accumulating science confirming the utility of focusing on writing about the body's functionality and/or expressing appreciation or gratitude for the body for enhancing aspects of positive body image [e.g., see (13) for a review] or reducing internalized weight bias in young adult (female) samples [e.g., (23, 74)]. We reasoned that this integrative approach would hold the potential to synergize the benefits of both practices with writing being able to reinforce participants' embodying experiences both on and off of the yoga mat through the processes of reflexivity and savoring. These self-regulatory processes are prospective mechanisms of effects that would benefit from further clarification in future research. Writing about one thing they were grateful their body enabled them to do each day may have also functioned as a way to motivate continued engagement with the weekly yoga practice and vice versa.

Nevertheless, in contrast to expectations, even with the targeted improvements witnessed in several aspects of positive body image and sizeable reduction in internal body shame, the intervention did not yield corresponding reductions in weight bias internalization, drive for leanness, or external body shame for members of the active experimental condition. Given that the Condition x Time interaction effects emerged as statistical trends in all three instances yet small-medium in magnitude suggests the promise of this intervention to impact these changes with further refinement. For instance, it is plausible that featuring a more intensive, structured writing prompt with examples more closely modeling prior research [e.g., (18, 23, 74)], recruiting a larger sample of young women with identified [e.g., (23)] or elevated weight bias internalization at baseline [e.g., (100)], extending the length of the intervention [e.g., (11)], and/or for example incorporating an explicit critique of diet culture and fat shaming at the sociocultural level [e.g., (102, 103)] may have proven useful in achieving this aim.

Moreover, despite the unique and timely contributions this early-phase trial provides to the extant literatures, it is noteworthy to acknowledge that there were no condition-specific gains found for self-compassion among the participants in the yoga and body gratitude writing condition. This was somewhat surprising given the seamless integration of compassion-focused lovingkindness meditation practices within the online yoga video content. At the same time, this study was not necessarily sufficiently powered to detect differences in more general experiences of self-compassion from a wait-list control [e.g., (72, 75, 101)] or an active control (77) as has been the focus of previous scholarship. A higher-powered study may further lend itself to a closer examination of the potential intervention effects on specific dimensions of the overall self-compassion construct. Meanwhile, increasing intervention length or other aspects of intensity in a larger sample may have led to greater targeted improvements. For example, Braun et al. (11) found that increases in self-compassion correlated with increases in intuitive eating in their 12-week mindful yoga program. Alternatively, perhaps including the domain-specific assessment of body compassion may have produced more targeted effects as a function of this brief online yoga-based intervention [e.g., (4)]. Similarly, unlike with respect to the other measures of positive body image assessed, the brief online yoga and body gratitude writing intervention did not produce significant condition-specific changes in body image flexibility over time. However, as visual inspection of the means suggests that they were moving in a favorable direction for the experimental group, it is again possible that a higher-powered study or integration of other design features that align with acceptance of body image-related internal events in the pursuit of valued action would yield a significant effect for this variable.

### Strengths, Limitations, and Future Directions

In addition to the strengths and innovations already noted above, this is among one of the more racially- and ethnically-diverse participant samples that has been featured in comparable yoga intervention science. We also attempted to minimize practical barriers to engaging with the yoga practice by providing yoga equipment kits to participants at the outset of the program which was largely appreciated by students. The pre-recorded yoga videos were all available online and archived using YouTube which not only increased accessibility to the content it also provided participants with the flexibility to practice at a time and location of their choosing which was more convenient for their schedules. Further, we viewed the inclusion of multiple components to the intervention as an asset which both served as a method of enhancing participant engagement and motivation and also may have mirrored more real-world conditions in which individuals may turn to multiple related resources to promote mindful self-care, positive embodiment, and well-being in their everyday lives (104). At the same time, there are several caveats to the current analysis that should be considered as a springboard for inspiring future research in this burgeoning area of scalable evidence-based, remotely-delivered wellness interventions for strengthening college student well-being.

Overall, though our exploratory results collectively are quite compelling, they should be interpreted somewhat cautiously given that the majority of analyses did not reach typical statistical power thresholds. Subsequent trials seeking to replicate and confirm the reliability of the observed effects in an adequately-powered sample are strongly encouraged. The large number of measures administered is a further caution as it may have resulted in the over-inflation of significant findings. It would also be of interest to explore whether framing the study in our recruitment materials explicitly as a yoga-based wellness promotion program (rather than as a “mind-body health promotion program”) may have impacted interest and who opted to complete our study's prescreener.

Reflecting on the limitations of the measurements implemented stimulates additional considerations for future research. For example, we used a measure of functional body awareness and appreciation that was originally developed and validated among first-time pregnant women (21) which may have impacted the internal consistency of one of the subscales we observed at baseline. Therefore, further psychometric evaluation of this measure in college-age or young adult, non-pregnant samples is warranted. Relatedly, these data were collected at a time that preceded the availability of the more commonly used *Functionality Appreciation Scale* [FAS; (4, 19)]. Another addition to prioritize in later-stage trials would be a more direct assessment of embodiment aligned with established theory [e.g., (27)] such as the *Experience of Embodiment Scale* [EES; (105)]. Further, it would be of interest to examine the extent to which this remotely-delivered home-based practice could be instrumental in fostering greater self-efficacy or intentions to participate in yoga in a studio or other external setting.

While the racial and ethnic diversity of the sample is a noted strength, it is possible that the inclusion of a larger-bodied White female yoga teacher may have been experienced as less relatable for some women of color from an intersectional perspective. Therefore, it would be important to further clarify the preferences of participants perhaps through incorporating more in-depth qualitative methodology (e.g., interviews or focus groups) and including additional dimensions of diversity that intersect with larger body size represented among the yoga teachers in designing later studies. Relatedly, we invite future research to prioritize inclusion of participants and yoga teachers reflective of a broader spectrum of diverse and intersecting social identities not necessarily represented here and who are typically underrepresented in typical yoga spaces and scholarship [e.g., males, gender non-conforming individuals, transgender individuals, sexual minorities, individuals living with physical disabilities, older adults, etc.: (60)]. Moreover, given the appeal of smartphone mobile applications among Gen Z and millennials, it would be beneficial to explore how barriers to uptake in practicing yoga may be further overcome using this technological modality among individuals in this target age group in subsequent research.

Additional methodological features to consider in future investigations involve: (1) incorporating an active control comparison condition to account for non-specific treatment effects, placebo effects, and demand effects [e.g., yoga without the body gratitude writing prompt; e.g., (23, 77)], (2) including a follow-up period to evaluate the maintenance of effects [e.g., (4, 10, 11, 23)], (3) using more objective measures of adherence to supplement self-report metrics, (4) further incentivizing completion of the daily body gratitude journaling component given the variable adherence observed (e.g., collecting the journals on a daily or weekly basis electronically with participants' consent, etc.), (5) exploring the sustainability of program access within the college's or university's existing infrastructure (e.g., offering for academic course credit or as part of the available student campus-based wellness resources), and (6) designing a dismantling study [e.g., (98)] guided by the Multiphase Optimization Strategy [MOST; (106)] and evaluated using the *Essential Properties of Yoga Questionnaire* [EPYQ; (107)] given the multiple intervention components involved. This last recommendation may yield insights into which particular component of the intervention is most influential in contributing to the observed effects. Arguably this may differ depending on variability in participants' experiences and preferences further adding to the complexity of nuances that may be relevant to consider in future intervention refinement. For example, in the present study, it appeared that the daily motivational images and yoga practices may have garnered greater appeal than wearing a motivational wristband or writing in the body gratitude journal among our participants who completed the program. Having a separate yoga only condition would also aid in assessing the potential additive benefit of the body gratitude journaling component which was precluded by the present study design.

Finally, it bears mentioning that one of the key limitations of prioritizing a per-protocol analysis to evaluate our exploratory secondary research questions is the likelihood of generating biased estimates in favor of individuals who sustained motivation to complete the study with minimal guidance and in the absence of live instruction. Therefore, we examined whether there were significant differences in baseline measures between participants who did and did not complete the study regardless of randomization condition. Notably, results suggested that individuals who score higher in drive for leanness and weight bias internalization along with reporting lower scores on functional body satisfaction and body image flexibility at baseline may be at increased risk for dropping out of the protocol. These findings provide insights that may help inform the design and inclusion criteria for future related research.

It is possible that an intervention with a higher level of structure and involvement by the research team (e.g., livestream classes, text message support) or even the inclusion of an online peer support component may be fruitful to explore in subsequent program development to address these individual difference needs {see (60) [Appearance Matters 9 Conference presentation] and (101) for novel examples of using a Canvas course website and a private Facebook group, respectively for this purpose}. Indeed, one of the tradeoffs of our program's format was that it was more individually-based and did not include the more traditional group-based experience of a more typical yoga class. This sense of community or sangha that can be forged over time in yoga practice spaces whether in-person or virtual is a critical element supportive of positive embodiment and wellbeing to consider incorporating in subsequent iterations of this intervention (68).

## Conclusion

The present pilot investigation provided preliminary evidence supporting the feasibility, acceptability, and per-protocol efficacy of a brief, online size-affirming yoga and body gratitude writing intervention among racially-diverse higher weight college women. Findings were particularly robust for changes in internal body shame and dimensions of positive body image. Our initial-stage analysis holds promise as a novel foundation for continuing to fuel advancements in the development and evaluation of scalable, virtually-delivered wellness promotion interventions that are aligned with bolstering efforts to realize the benefits of yoga on positive embodiment for all bodies. Importantly, further contributing to this mission has become even more urgent in strengthening health and wellbeing in the post-COVID-19 era.

## Data Availability Statement

The raw data supporting the conclusions of this article will be made available by the authors, without undue reservation.

## Ethics Statement

The studies involving human participants were reviewed and approved by UNC Charlotte IRB. The patients/participants provided their written informed consent to participate in this study.

## Author Contributions

JW served as the lead PI and additionally contributed to conceptualizing the design of the study and to carrying out the data analysis plan. All authors have contributed to the process of manuscript development and review.

## Funding

This study received funding support from the UNC Charlotte Faculty Research Grant and the UNC Charlotte Department of Psychological Science SEED Grant. Publication financial support was received from the UNC Charlotte College of Liberal Arts and Sciences.

## Conflict of Interest

All authors declare that the research was conducted in the absence of any commercial or financial relationships that could be construed as a potential conflict of interest.

## Publisher's Note

All claims expressed in this article are solely those of the authors and do not necessarily represent those of their affiliated organizations, or those of the publisher, the editors and the reviewers. Any product that may be evaluated in this article, or claim that may be made by its manufacturer, is not guaranteed or endorsed by the publisher.
